# Targeting the Mitochondria in High-Grade Gliomas

**DOI:** 10.3390/cancers17183062

**Published:** 2025-09-19

**Authors:** Shaunak Sathe, Qi Li, Jinkyu Jung, Jing Wu

**Affiliations:** Neuro-Oncology Branch, Center for Cancer Research, National Cancer Institute, Bethesda, MD 20892, USA; sathesm@nih.gov (S.S.); qi.li2@nih.gov (Q.L.); jinkyu.jung@nih.gov (J.J.)

**Keywords:** high-grade glioma, glioblastoma, DMG, mitochondria, clinical trial

## Abstract

High-grade gliomas are aggressive primary brain tumors and often fatal. They are characterized by rapid growth, treatment resistance, and significant heterogeneity both within and between tumors. A growing body of evidence highlights the mitochondria, dynamic organelles essential for energy production, apoptosis regulation, and metabolic rewiring, as a critical driver in glioma progression and treatment resistance. As a result, these insights have sparked growing interest in mitochondrial-directed therapies. This review highlights the distinct metabolic features and mitochondrial processes of glioma, outlining the rationale for targeting mitochondrial function. We discuss recent advances in mitochondrial-targeted therapies, with a focus on caseinolytic protease P (ClpP) agonism as a breakthrough in the treatment of diffuse midline glioma (DMG). Moreover, we discuss the pathogenic link between mitochondrial metabolism and epigenetic regulation, and the potential therapeutic benefit of disrupting this interaction.

## Simple Summary

High-grade gliomas are aggressive brain tumors associated with poor outcomes. Mitochondria play an essential role in glioma growth and resistance to therapy, making them a promising target for novel therapeutic strategies. This review summarizes the mitochondrial dysfunctions that drive glioma progression, highlights clinical trials and therapies aimed at mitochondrial targeting, and discusses emerging approaches such as caseinolytic protease P (ClpP) and disruption of mitochondrial–epigenetic interactions that may improve outcomes for patients with high-grade glioma.

## 1. Introduction

High-grade gliomas are malignant and incurable brain tumors, leading to significant morbidity and mortality in both adults and children [1]. The World Health Organization (WHO) classifies high-grade gliomas as either grade 3 or grade 4tumors, based on histopathological criteria [2]. Among these, glioblastoma and diffuse midline glioma (DMG) represent some of the most aggressive and lethal diseases.

Glioblastoma accounts for the majority of malignant gliomas and is diagnosed in approximately 3.27 per 100,000 people/year [1]. For the past two decades, the standard of care for newly diagnosed glioblastoma has remained initial maximal safe resection followed by radiation with concomitant and adjuvant temozolomide (TMZ)—an alkylating agent that induces DNA damage [3]. Despite this aggressive treatment, patients have a median survival of only around 14–20 months after initial diagnosis. Glioblastoma almost always develops resistance and recurs within 7 to 9 months, with rapid progression leading to an incredibly dismal prognosis [4].

Diffuse midline glioma (DMG) is a type of high-grade brain tumor that primarily affects children and young adults. It is the most lethal childhood cancer, with a median overall survival of 9–11 months and a 2-year survival of less than 10%, despite six decades of intense conventional and experimental therapies that have been unable to significantly improve patient outcomes [5,6]. DMG is now a broader term that includes tumors with similar genetic characteristics, particularly histone mutations such as H3K27M, located along the midline structures of the brain [2]. Critically, these mutations lead to hypomethylation of lysine 27 of histone H3 (H3K27), which is believed to drive an epigenetic phenotype that promotes aberrant expression of oncogenes resulting in rapid tumor growth. Over 80% of DMG cases exhibit the missense mutation H3K27M that leads to this hypomethylation [2,7].

Although extensive research efforts have provided exciting insight into the biochemical mechanisms that drive high-grade gliomas, clinical outcomes have improved only modestly, and both glioblastoma and DMG remain universally fatal.

Poor prognosis of these tumors can be attributed to rapid progression and resistance to chemotherapy and radiation. Growing evidence implicates the mitochondria as a key driver of these therapeutic challenges. In glioma, as well as breast, colorectal, lung, pancreatic and several other tumors, mitochondrial dysfunction has been identified as a contributor to tumor growth and therapy resistance, establishing the mitochondria as a common cancer driver [8,9,10,11,12]. These insights have led us and others to rationalize targeting the mitochondria as a promising therapeutic strategy for glioma [13].

Here, we begin by highlighting the distinct metabolic properties of glioma and focus on the mitochondria as a critical driver of metabolic rewiring. We discuss how aberrant mitochondrial quality control contributes to treatment resistance and glioma progression and highlight the mitochondria as an attractive therapeutic target. Finally, we provide a summary of mitochondrial targeting efforts, including a therapeutic breakthrough through ClpP agonism, and touch on crosstalk between mitochondrial metabolism and epigenetics that may be exploitable for glioma treatment.

## 2. Distinct Metabolic Properties of Glioma

Gliomas undergo extensive metabolic reprogramming to support their aggressive growth, acquiring distinct metabolic properties. Mitochondria are as central regulators of this reprogramming, with their functional state influencing both energy production and nutrient uptake. Critical metabolic processes, including fatty acid β-oxidation and the tricarboxylic acid (TCA) cycle take place within the mitochondrial matrix, highlighting the organelle’s essential role in glioma metabolic adaption [14,15].

### 2.1. The Warburg Effect

The first observation of reprogramming of cancer metabolism came from a landmark study in cancer biology by Warburg et al., which was later termed the Warburg effect— that cancer cells upregulate “aerobic glycolysis” by taking up high amounts of glucose to produce lactate regardless of the presence of oxygen [16]. The Warburg effect is well-described as a hallmark in glioma and a key part of the fine-tuned metabolic reprogramming that drives the survival and proliferation of glioma cells. This process seems counterintuitive and energetically inefficient, generating only a net of 2 ATP molecules per glucose compared to up to 38 ATP molecules produced during oxidative phosphorylation (OXPHOS) [17].

So, why would cancer cells prefer this energetically inefficient process? Discussion has centered on four main arguments. First, the Warburg effect reprograms cancer cells to improve access to a limited resource (glucose) for energy production to meet their needs for rapid proliferation. Secondly, it promotes energy flux into several biosynthetic pathways to produce vital cell building blocks, such as nucleotides, amino acids, and lipids. Thirdly, it acidifies the tumor microenvironment (TME) to disrupt tissue architecture and cause immune suppression. Lastly, it allows for cell signaling alterations due to changes in reactive oxygen species (ROS) and chromatin modulation [18]. While the benefit of the Warburg effect remains a highly disputed topic, the literature has supported a general explanation: the Warburg effect enables cancer cells to uptake and incorporate nutrients into the biomass (nucleotides, amino acids, and lipids) needed to produce a new cell [19].

The origin of extensive metabolic reprograming is driven, at least in part, by the intrinsic hypoxic properties of many primary brain tumors. Malignant gliomas cells are known to accumulate hypoxia-inducible factors (HIFs), acting as hypoxia sensors that orchestrate a coordinated response to increase their pro-survival and pro-invasive phenotype [20,21].

Metabolic rewiring in glioma is also evidenced by the upregulation in glucose uptake, addiction to glutamine, increased fatty acid synthesis and beta-oxidation, and the alterations of the TCA cycle [17,22,23,24,25].

### 2.2. Increased Reliance on Glucose and Glutamine

To support the need for glycolytic activity, glioma cells highly upregulate the expression of glucose-transporters, GLUT1 and GLUT3, enhancing glycolytic flux into the cell [22]. Astrocytic end-feet envelop capillaries in the blood–brain barrier, allowing malignant cells to tightly upregulate glucose influx [26]. Several studies have reported that increased expression of GLUT1 and GLUT3 is linked to worse prognosis and higher-grade tumors [22,27,28]. GLUT3 has been linked to tumor cell invasion in glioblastoma [28]. With increased glucose uptake, a pool of metabolites can then be shuttled to increase intracellular lipid synthesis, amino acid, and nucleotide stores to meet the cell’s mitotic needs [17].

In addition to glucose, glioma cells strongly rely on glutamine for fuel for several reasons. Glutamine serves as a critical anaplerotic substrate, replenishing TCA cycle intermediates in the mitochondria through its conversion to glutamate and subsequently to α-ketoglutarate. Glutamine metabolism also contributes to redox homeostasis by supporting the production of glutathione, a key antioxidant that helps glioma cells resist oxidative stress [23,29]. These metabolic adaptations are often driven by oncogenic signaling, including c-Myc-mediated upregulation of glutaminase and mutations in genes such as EGFR, which further enhance glutamine utilization to support tumor growth [30].

### 2.3. Upregulated Beta-Oxidation and Fatty Acid Synthesis

Fatty acid synthesis is generally upregulated in glioma, with fatty acid synthase expression correlating with higher tumor grades. Various studies have attributed this upregulation of fatty acid synthesis to the activity of sterol response element binding protein (SREBP), a transcription factor [24,30,31,32]. Fatty acids have various uses for tumor cells, such as oncogenic signaling molecules, or geared toward building new lipid bilayers and vacuolar membranes to meet the cell’s needs [25].

Fatty acid oxidation (FAO) is also upregulated in glioma, particularly in glioblastoma, providing a critical alternative energy source. Several studies have linked increased FAO to therapy resistance and survival in glioblastoma [25,33,34]. FAO is required for the respiration and proliferation of glioblastoma cells, and FAO enzymes have demonstrated potential as prognostic markers [34]. Enhanced FAO in glioblastoma was also shown to be directly dependent upon the TME, leading to a metabolic plasticity that allows glioma cells to adapt and proliferate [33].

### 2.4. Altered TCA Cycle Function

Glioma cells display highly altered TCA cycle function and maintain the TCA cycle under hypoxic conditions through replenishing α-ketoglutarate, by taking up glutamine and aspartate from their environment [35]. In high-grade gliomas, intermediates of the TCA cycle are shuttled into various biosynthetic pathways to fuel tumor growth. For example, citrate can be diverted into fatty acid synthesis while α-ketoglutarate can be shuttled into amino acid synthesis [35,36].

Isocitrate dehydrogenase (IDH) enzymes are key players in the TCA cycle, facilitating the conversion of isocitrate into α-ketoglutarate while using NADP^+^ or NAD^+^ as cofactors. While the IDH2 enzyme resides in the mitochondria and is more directly involved in the TCA cycle, IDH1 resides in the cytoplasm and is involved in redox homeostasis [37]. In glioblastoma, the expression of wild-type IDH1 is increased about four times relative to healthy brain tissue [38]. This makes IDH1 the major source of NADPH, a compound known to fuel antioxidant defenses and protect tumor cells from oxidative damage. Knockdown of IDH1 depletes NADPH levels and sensitizes glioblastoma cells to radiation, leading to cellular senescence [37,38].

Although IDH mutations are uncommon in gliomas diagnosed de novo as high-grade, they are present in approximately 70–80% of low-grade gliomas. Importantly, most IDH-mutant, low-grade gliomas eventually undergo malignant transformation into high-grade tumors. As a result, IDH mutation remains a defining metabolic alteration in a biologically distinct subset of high-grade gliomas [39,40,41].

Glioma cells exhibit extensive metabolic rewiring to support tumor growth, as exemplified by the Warburg effect and other altered metabolic pathways. Dysfunctional mitochondria play a central role in regulating these processes and have emerged as a promising focus of research [14]. Targeting mitochondrial-related metabolism has revealed novel therapeutic vulnerabilities. For instance, inhibiting glutaminolysis in mitochondrial-dependent glioblastoma cells disrupted TCA cycle function and energy production, leading to impaired tumor cell survival [42]. In addition to these metabolic dependencies, dysregulation of mitochondrial quality control has also been implicated in glioma progression.

## 3. Aberrant Mitochondrial Quality Control in Glioma

**Figure 1:**
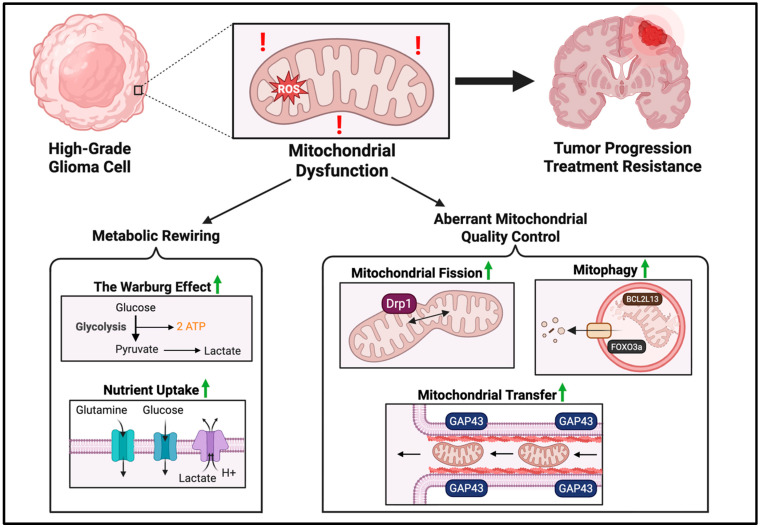
Mitochondrial dysfunction drives glioma progression and treatment resistance. Mitochondrial dysfunction involves both metabolic rewiring and aberrant quality control. Metabolic rewiring is characterized by the Warburg effect, a reliance on glycolysis even in the presence of oxygen. To meet metabolic demands, glioma cells upregulate glucose and glutamine influx, while removing excess lactate. Mitochondrial quality is controlled in several ways: mitochondrial transfer from adjacent cells via tunneling actin nanotubules, upregulation of mitochondrial fission, and general upregulation of mitophagy levels. Red exclamation symbols are used to represent mitochondrial dysfunction. Green arrows are used to represent upregulation. The image was created with BioRender.com (accessed on 25 August 2025).

Glioma cells exhibit aberrant mitochondrial processes that contribute to tumor progression and therapy resistance. As summarized in Figure 1, processes such as mitochondrial transfer, mitophagy, mitochondrial fusion and fission, are highly altered in glioma in order to control mitochondrial quality.

### 3.1. Mitochondrial Transfer

One alteration of mitochondrial processes in glioma progression is through a process known as mitochondrial transfer, to import mitochondria from and to adjacent cells via tunneling actin nanotubes. Mesenchymal stem cells were shown to transfer mitochondria to recipient glioblastoma cells, inducing metabolic and functional changes [43]. This transfer leads to a key metabolic shift from glucose to glutamine utilization in glioma stem cells (GSCs), accompanied by a higher orotate turnover and increased nucleotide synthesis, contributing to increased TMZ resistance in glioblastoma cells [43]. Watson et al. similarly reported a high prevalence of mitochondrial transfer from adjacent healthy astrocytes, increasing mitochondrial respiration and upregulating metabolic pathways linked to proliferation and tumorigenicity. This mitochondria transfer is facilitated by growth-associated protein 43 (GAP43), involved in neuron axon regeneration and astrocyte reactivity [44]. Further studies have also suggested a role of mitochondrial transfer in creating an immunosuppressive TME. Cancer cells can transfer mitochondria containing mutated mtDNA to tumor-infiltrating lymphocytes, reducing their energy production and impairing their antitumor activity [45]. Mitochondrial transfer appears to be an important process in glioma progression and remains a highly unexplored yet potentially promising area of research.

### 3.2. Upregulation of Mitophagy

The cellular process of mitophagy, the cellular breakdown and recycling of damaged mitochondria, is generally upregulated in glioma cells as a protective mechanism to deal with cellular stressors. Prognostic models using a mitophagy-related gene signature have shown remarkable accuracy in predicting overall survival, demonstrating the clinical relevance of mitophagy in glioma patients [46,47]. In particular, the BCL2L13 protein has been shown to promote mitochondrial fission-dependent protective mitophagy in glioblastoma cells [48]. Moreover, the induction of mitophagy by FOXO3a has been reported to promote cell survival by protecting glioma cells from temozolomide-induced cytotoxicity [49]. In high-grade glioma cells, mitochondrial respiratory cristae are stabilized and remodeled through mitophagy, enhancing OXPHOS and ultimately promoting aggressive disease progression [50]. While this evidence is strong, it has also been reported that cannabidiol treatment induces high mitophagy levels to kill glioma cells [51]. Thus, the role of mitophagy in tumor progression remains to be elucidated and may function as a double-edged sword.

### 3.3. Mitochondrial Dynamics

Glioma mitochondria also have altered mitochondrial dynamics—including fission and fusion—which are reported to be critical in glioma progression [52]. Mitochondrial fusion can allow gene products to be transferred between mitochondria for optimized function, especially under metabolic and environmental stresses. Mitochondrial fission is essential for mitochondrial division and quality control [14]. In glioma stem cells, which are responsible for tumor initiation and maintenance, mitochondrial fission is upregulated, along with its key regulator, dynamin-related protein 1 (Drp1), promoting increased cell migration and invasiveness [53]. Drp1 has also been shown to cause hypoxia-induced glioblastoma cell migration [54]. Increased mitochondrial fission and decreased mitochondrial fusion seem to contribute to tumorigenicity in glioma, with Drp1 emerging as a potential therapeutic target. Shifting of mitochondrial dynamics maintains mitochondrial quality and likely reflects the changing needs of the tumor cells throughout tumor progression.

Aberrant mitochondrial quality control and extensive metabolic reprogramming in glioma highlight the mitochondria as critical in tumor progression and treatment resistance. Notably, emerging literature suggests that targeting the mitochondria in glioma may help overcome longstanding therapeutic challenges [14,15,55,56].

## 4. Therapeutic Challenges in Glioma and the Promise of Mitochondrial Targeting

The challenges to treating glioma are multifold, resulting in difficulties in improving the standard of care. As previously discussed, glioma cells are dynamic and can be fueled through multiple metabolic pathways. The disease is also rapidly evolving, supported by self-renewing GSCs, protected by the blood–brain barrier (BBB) and the blood–tumor barrier (BTB), which are difficult to penetrate, and located within an immunosuppressive tumor microenvironment. Complicating therapy further, high-grade gliomas are characterized by incredibly high level of heterogeneity [57].

### 4.1. High Inter- and Intratumor Heterogeneity

A major challenge to treatment of high-grade gliomas, particularly glioblastoma, is the high intra- and intertumoral heterogeneity [58]. Characterizing intratumor heterogeneity through anatomical features, the Ivy Glioblastoma Atlas Project has mapped five key components within tumors: leading edge, tumor-infiltrating, cellular tumor, microvascular proliferation, and pseudo palisading cells around necrosis. These regions can be further categorized into specialized niches: perivascular (microvascular proliferation), hypoxia–necrotic (cellular tumor, pseudo palisading cells around necrosis), and invasive (leading edge and tumor-infiltrating) [59]. With each having varied metabolic dependencies, targeted therapies often only affect a certain subset of glioma cells, while residual niches may survive and cause recurrence. Thus, there is an unmet need for therapies that more universally target malignant glioma cells.

Intertumor heterogeneity also presents as a major challenge to treating glioma. Various glioblastoma subtypes between different tumors can be mapped along neurodevelopmental and metabolic axes, creating 4 general classifications: proliferative/progenitor, neuronal, glycolytic/plurimetabolic and mitochondrial [60].

### 4.2. Targeting the Mitochondria in Subtypes of High-Grade Gliomas

In the past, clinical trials for mitochondrial inhibitors have generally lacked distinction between different metabolic subtypes in high-grade gliomas. Future clinical trials may benefit from a more precise approach by targeting tumors with a higher mitochondrial dependency. Emerging literature has corroborated the efficacy of a precision medicine approach, utilizing mitochondrial-targeting therapies directed at high-grade gliomas that exhibit higher OXPHOS dependence. It was reported that mitochondrial, but not glycolytic/plurimetabolic glioblastoma, exhibited remarkable vulnerability to inhibitors of OXPHOS. Interestingly, the mitochondrial subtype showed higher frequency of CDK4/MDM2 amplification, and all NRAS-mutated glioblastomas mapped exclusively to this subtype, suggesting potential clinical markers. With over 20% of glioblastomas fueled by mitochondrial hyperactivity, this targeted strategy seems promising [60]. In addition, the mitochondrial energy output through OXPHOS has been identified as an essential factor for the rapid cellular proliferation of diffuse midline glioma (DMG) [61]. Thus, DMGs are also believed to be particularly vulnerable to the mitochondrial-targeting strategy, as discussed in detail in a later section.

Notably, while this strategy may be most effective in high-grade gliomas that are especially reliant on the mitochondria, there is evidence that mitochondrial targeting still has a broader effect across diverse glioma niches and subtypes. Given the high metabolic heterogeneity within a tumor, there is evidence that even glycolytic glioma cells can respond to mitochondrial targeting. Though these cells rely on glycolysis for energy production during early stages, lactic acid buildup progressively reduces glycolysis levels and significantly increases OXPHOS processes (approximately 2–4 times) throughout disease progression to generate adequate energy, rendering them vulnerable to OXPHOS inhibitors [62,63]. It is also reported that self-renewing GSCs, which are thought to initiate disease recurrence and treatment resistance, strongly rely on OXPHOS more than differentiated cells [64]. Additionally, it has been demonstrated that glioma cells do, in fact, contain intact energy-producing mitochondria with normal overall OXPHOS levels even while glycolysis remains upregulated [65,66]. Thus, a mitochondrial targeting strategy may indeed have a more wide-ranging efficacy across glioma subtypes and intertumoral niches.

With this strong rationale for mitochondrial targeting, several compounds have been developed to impair mitochondrial functioning in glioma, attempting to exploit vulnerabilities into clinically meaningful outcomes.

## 5. Efforts in Targeting the Mitochondria in Glioma

Mitochondrial targeting encompasses a large umbrella of drugs that inhibit various mitochondrial processes.

### 5.1. Targeting the Mitochondria Through OXPHOS Inhibitors

Targeting of the Electron Transport Chain (ETC) through OXPHOS inhibitors has emerged as an effective therapeutic strategy. The ETC, located in the inner mitochondrial membrane is made up of five complexes. Complexes I–IV are involved in the transfer of electrons from electron carriers (NADH and FADH2) to oxygen via oxidation, coupled with the pumping of protons into the intermembrane space. Complex V, known as ATP synthase, then utilizes this electrochemical gradient to phosphorylate ADP into ATP—known as oxidative phosphorylation [67,68]. The ETC is also involved in the production of reactive oxygen species (ROS), which function as somewhat of a double-edged sword in the cell. While moderate levels of ROS are generally associated with genomic instability and tumorigenicity, highly dysregulated ETC function can to a large buildup of ROS resulting in apoptosis [69,70]. In general, OXPHOS inhibitors work by interfering with the ETC to deplete energy stores and increase ROS accumulation.

#### 5.1.1. Inhibitors of Complex I

Complex I (NADH ubiquinone–oxidoreductase) inhibitors have shown promise in preclinical and clinical trials for glioma. Metformin, a biguanide used as an anti-diabetic drug, is known to inhibit Complex I and impair OXPHOS [71,72]. Metformin has been investigated with mefloquine, memantine and TMZ in glioblastoma patients after radiation treatment in a phase II study that suggests effectiveness (median survival of 21 months with a 2-year OS of 43%) and a safety profile of the combined treatments [73]. However, more recently, a phase 2 trial (KNOG-1501 study) demonstrated that while the metformin plus temozolomide regimen was well tolerated, it did not confer a clinical benefit in patients with recurrent glioblastoma [74]. Still, various clinical trials are ongoing seeking to repurpose metformin to treat glioma, and the practicality of the therapy remains to be elucidated [75,76,77].

IACS-010759 is an incredibly selective and potent small-molecule complex I inhibitor, which was advanced into two dose-escalation phase I trials in patients with relapsed/refractory acute myeloid leukemia (NCT02882321, *n* = 17) and advanced solid tumors (NCT03291938, *n* = 23) [78]. Despite preclinical evidence of efficacy, only modest antitumor activity was observed at tolerated doses [78]. Intolerable neurotoxicity precluded adequate dosing and prevented the trial from continuing.

IM156, another biguanide and Complex I inhibitor, showed more promise [79]. Preclinical evidence in glioblastoma demonstrated a potent antitumor effect. In a first-in-human phase I study in patients with advanced solid tumors, IM156 was tolerated and was the first OXPHOS inhibitor to establish a recommended phase II dose (RP2D) for further clinical development in cancer. Adverse events were manageable, and stable disease for around 30% of patients with advanced solid tumors was the best reported response [79].

#### 5.1.2. Inhibitors of Complex II

While complex II inhibition has not been investigated in clinical trials for glioma, several complex II inhibitors have demonstrated preclinical efficacy. The atypical adamantyl retinoid, ST1926, alleviated mitochondria-regulated bioenergetics in glioma cells via reducing ATP production and promoting ROS production [80]. The Complex I and II inhibitor and Vitamin E derivative, α-tocopheryl succinate, was reported to potentiate the response to etoposide, a topoisomerase inhibitor, in multidrug-resistant glioblastoma cells [81]. Gracillin, a selective Complex II inhibitor, exhibited potent energy depletion and induced apoptosis in a broad spectrum of cancer cell lines [82]. However, low BBB permeability of gracillin seems to limit its practicality in glioma treatment [82].

#### 5.1.3. Inhibitors of Complex III

Targeting Complex III has also demonstrated preclinical efficacy in gliomas, including drugs such as Mahanine, Atovaquone, Antimycin A, and Licochalcone A. Mahanine, a plant-derived Complex III inhibitor, leads to cell cycle arrest and DNA damage response in glioblastoma cell lines. The accumulation of ROS resulted in Chk1/Chk2 upregulation and activation, which are essential factors for G0/G1 arrest [83,84]. Atovaquone demonstrated cytotoxicity against glioblastoma cell lines as well as provided a confirmed target for atovaquone brain concentrations in in vitro cell viability studies. Due to limitations in the bioavailability of atovaquone, enhanced amorphous solid dispersion was utilized as a proof-of-concept to deliver therapeutically effective brain levels of atovaquone for the treatment of glioblastoma [85]. Antimycin A demonstrated efficacy in treating glioblastoma cell lines when combined with the antimetabolite, 3-bromopyruvate, to critically increase ROS levels and induce cell death [86]. Licochalcone A, a natural chalconoid from licorice root, induced massive caspase-dependent death in GSCs through mitochondrial fragmentation and reduced ATP production [87]. Overall, targeting Complex III and the resulting ROS elevation may prove a promising therapeutic strategy though yet to be proven in a clinical setting.

#### 5.1.4. Inhibitors of Complex IV

Complex IV inhibitors, such as arsenic trioxide (ATO), have shown promising anti-glioma effects through induction of autophagy and apoptosis in GSCs. In both in vitro and in vivo glioblastoma models, arsenic trioxide induced cell death through downregulation of survivin [88]. Several clinical trials have evaluated the efficacy of arsenic trioxide. A phase II clinical trial reported that arsenic trioxide did not improve overall survival in glioblastoma patients compared to historical data [89]. In contrast, a phase I clinical trial (NCT00045565) reported that arsenic trioxide with standard radiation is well tolerated in patients with newly diagnosed glioblastoma. Even without TMZ or adjuvant therapy, the overall survival of all patients (17.7 months) and especially patients who received biweekly arsenic trioxide (22.8 months) was promising [90]. It is important to note that while being a Complex IV inhibitor, arsenic trioxide has several off-target effects that contribute to its antitumor efficacy [91]. The practicality of this therapy in glioma still remains to be elucidated.

#### 5.1.5. Inhibitors of Complex V

Targeting ATP Synthase (Complex V) has emerged as an exciting therapeutic vulnerability, corroborated by strong preclinical evidence. Gboxin, a small-molecule OXPHOS inhibitor, specifically targeted mouse and human glioblastoma cells but not that of mouse embryonic fibroblasts or neonatal astrocytes [92]. Due to limitations in bioavailability, a biomimetic Gboxin nanomedicine was developed that demonstrated good biocompatibility, improved pharmacokinetic profile, efficient BBB permeability and homotypic dual tumor cell and mitochondria targeting [93]. This established preclinical efficacy led to an ongoing Phase I clinical trial to evaluate S-Gboxin, in addition to standard TMZ/radiation therapy for glioblastoma and diffuse midline glioma (NCT06806228). Other Complex V inhibitors, such as bedaquiline and leucinostatin, have demonstrated ability to target ATP synthase, though their efficacy in glioma is unclear [94,95].

A summary of clinical trials in glioma that employ OXPHOS inhibitors is provided in Table 1.

### 5.2. Additional Mitochondrial Targeted Strategies in Glioma

While several OXPHOS inhibitors targeting the mitochondrial respiratory complexes have advanced to clinical trials for glioma, other mitochondrial-targeting strategies including inhibition of β-oxidation and disruption of mitochondrial matrix functions have demonstrated preclinical efficacy. Few of these approaches have advanced to the clinical trial stage.

#### 5.2.1. Inhibitors of Beta-Oxidation

Targeting fatty acid oxidation has emerged as a promising therapeutic vulnerability. Etomoxir is a carnitine palmitoyltransferase I inhibitor that prevents ß-oxidation of fatty acids to reduce energy levels. Etoximir was shown to reduce energy production and cellular proliferation in glioma cells [34]. Interestingly, in patient-derived glioblastoma tumor spheres, etomoxir led to a lethal energy reduction, which was exacerbated in combination with TMZ [98]. Etomoxir also effectively reduced invasiveness and improved survival in xenograft glioblastoma mice models [98]. An apparent limitation, however, is that etomoxir was found to have cross reactivity with other proteins, leading to further targets beyond solely fatty acid oxidation [99]. To our knowledge, etomoxir has not yet been translated into clinical trials.

#### 5.2.2. Compounds Targeting Mitochondrial Matrix

Devimistat or CPI-613 selectively targets the TCA cycle enzymes, pyruvate dehydrogenase and α-ketoglutarate dehydrogenase, in order to compromise mitochondrial processes. This interference initiates apoptosis in glioblastoma cell lines and animal models, especially when combined with a BH3-mimetic [55,100,101]. CPI-613 achieved promising outcomes in phase I clinical trials in pancreatic cancer, with treatment being well-tolerated [102].

Gamitrinib, also known as geldanamycin, is a mitochondrial matrix inhibitor that was reported to inhibit of proliferation and induce apoptosis in several glioma cell lines, patient-derived organoids, and xenograft models [103]. This antitumor effect is also further potentiated when combined with a BH3-mimetic. Gamitrinib is currently undergoing assessment in a phase I clinical trial (NCT04827810) involving patients with advanced malignancies, including glioblastoma [104].

### 5.3. Effects of Mitochondrial Targeting on Cancer Signaling Networks

Mitochondrial targeting also activates several relevant signaling cascades. Many mitochondrial inhibitors lead to accumulation of ROS, which can overwhelm antioxidant defenses and disrupt key survival pathways, ultimately inducing cytotoxicity in glioma cells [86,105]. For example, the phytochemicals, celastrol and triptolide, significantly reduced mitochondrial membrane potential and increased ROS levels, thereby activating the ROS/JNK pathway and triggering autophagy and apoptosis in glioma cells [106,107]. Additionally, ROS accumulation has been shown to inhibit the PI3K/Akt/mTOR pathway, a critical regulator of cancer cell survival and therapy resistance [108]. Together, these findings underscore the therapeutic potential of elevating mitochondrial ROS to disrupt oncogenic signaling and suppress glioma progression.

Interestingly, it has also been shown that targeting mitochondrial function and OXPHOS can destabilize hypoxia-inducible factor 1-alpha (HIF-1α), a critical regulator of glioma progression under hypoxic conditions [109]. Pharmacological inhibition of OXPHOS with metformin or rotenone resulted in decreased HIF-1α expression levels in cancer cells, as well as decreased carbonic anhydrase IX and vascular endothelial growth factor levels, key drivers of invasiveness and angiogenesis [109].

Mitochondrial-targeting drugs can also disrupt mitochondrial membrane potential and cause structural damage. One key effect is outer membrane permeabilization (MOMP), often mediated by BH3-only BCL-2 proteins. This permeabilization facilitates the release of cytochrome c into the cytosol, leading to activation of caspases and initiation of the intrinsic apoptotic pathway in glioma cells [110,111]. These signaling cascades highlight the broad cellular impact of mitochondrial-targeting strategies, which aim to exploit redox balance and disrupt oncogenic pathways to induce potent antitumor responses.

## 6. Breakthrough in Targeting Mitochondrial Function: Imipridones Induce Mitochondrial Dysfunction in DMG via Hyperactivation of Caseinolytic Protease P

A new generation of mitochondrial-targeting compounds called imipridones has recently offered a glimmer of hope, particularly in the treatment of diffuse midline glioma (DMG). Imipridones are highlighted here, as they have demonstrated strong clinical potential in a disease that has seen limited therapeutic progress over the past several decades.

### 6.1. Imipridones, a New Drug Class with Potent Antitumor Effects

The first generation of imipridones, ONC201, was originally discovered as TNF-Related Apoptosis Inducing Ligand (TRAIL)-inducing compound 10, in a small-molecule screen of colorectal cancer cell lines [112]. It was reported to activate the integrated stress response, an adaptational signaling pathway that allows cells to shut down protein synthesis through ATF4/CHOP. This cascade ultimately causes cell death through endoplasmic reticulum stress and upregulation of TRAIL receptor DR5 [113].

Using a Bayesian machine learning approach, ONC201 was originally identified as a dopamine receptor D2 (DRD2) antagonist, with in silico modeling confirming ONC201 binds to the active site of DRD2 [114,115]. As DRD2 was previously reported as expressed across brain tumor types, and is linked to its metabolic plasticity, this antagonism was promising [116,117]. In several studies, the antagonism of DRD2 by imipridones was reported to inhibit cell proliferation, induce cell cycle G1 arrest, reduce cell invasion, and cause apoptosis [118]. Despite this, recent evidence has suggested that DRD2 antagonism cannot fully explain the antitumor effect of imipridones. Cancer cells with limited expression of DRD2 still remain sensitive to ONC201, suggesting alternative mechanisms of action [119,120]. Transient knockdown of DRD2 in colorectal cancer cells minimally affected response to ONC201, further indicating DRD2 antagonism may not be the cause of imipridone cytotoxicity [121].

### 6.2. ClpP Identified as a Target of Imipridones

Recent studies have attributed the antitumor effect of imipridones to agonism of the caseinolytic protease P (ClpP), a serine protease located specifically in the mitochondrial matrix that regulates protein integrity and several key mitochondrial functions. ClpP forms a heterodimer with its ClpX chaperone, creating a complex termed ClpXP. When functioning normally, ClpXP performs protein quality control in the mitochondria by degrading denatured or misfolded protein to maintain the integrity of the respiratory chain and sustain OXPHOS. Mechanistically, ClpP agonists displace ClpX, opening the pore of the ClpP protease and strongly increasing its protease activity leading to mitochondrial degradation [122].

ClpP was confirmed as a target of imipridones through crystallography and biochemical studies, and ClpP agonism was shown to induce selective lethality in cancer cells [119,120]. In silico binding of ONC201 to ClpP also confirmed ClpP as a target [115]. Furthermore, CRISPR-mediated ClpP knockdown in DMG cell lines abolished ONC201 efficacy, supporting ClpP as a critical mediator of its antitumor activity [123]. As ClpP is overexpressed across diffuse gliomas at an mRNA and protein level, the therapeutic potential of imipridones in glioma remains incredibly promising [124,125,126].

Moreover, investigators have recently developed a new class of more potent and selective imipridones, known as TR compounds [120]. These compounds were reported to be solely ClpP agonists without DRD2 antagonist function. Several preclinical studies so far have reported potent efficacy of TR-107 and related analogs in breast, colorectal, and brain tumor models [127,128,129]. Imipridone treatment and ClpP agonism hyperactivated mitochondrial proteolysis, leading to the uncontrolled degradation of essential mitochondrial proteins, disruption of OXPHOS, catastrophic metabolic collapse, and ultimately cell death [115,119,122]. In glioblastoma, combining TRAIL-secreting neural stem cells with TR-107 synergistically activated apoptotic caspases, overcame TRAIL resistance, suppressed tumor growth, and significantly extended survival in mouse xenograft models [129]. While imipridone therapy has been tested in several clinical trials across a variety of cancers, it has demonstrated specific clinical utility in H3K27-altered diffuse midline glioma.

### 6.3. Clinical Efficacy of Imipridones for Diffuse Midline Glioma

Several clinical trials have evaluated the safety and efficacy of imipridones, including ONC201 and its more potent analog ONC206, in diffuse gliomas. A first-in-human clinical trial of ONC201 (NCT02525692) for diffuse gliomas was opened in August 2015 and was later expanded to include H3K27-altered DMG patients after an exceptional responder experienced a near-complete objective response including regression of the primary thalamic region [130,131]. Various clinical trials have opened in recent years to evaluate the efficacy of imipridones in DMG. A phase I dose escalation and an expansion trial (NCT03416530 and NCT03134131) of ONC201 reported aggregate results, with a median overall survival of patients with non-recurrent H3K27M DMG (*n* = 35) of 21.7 months from diagnosis and 9.3 months at recurrence [132]. The trials reported no dose-limiting toxicities, with a treatment dose that was overall well tolerated [132]. These incredibly promising results have led to several ongoing Phase 2 and 3 trials, and ONC201 is expected to be submitted for accelerated FDA approval by the end of the year.

### 6.4. Evaluating the Response to Imipridone Therapy

To monitor treatment response, recent studies have searched for prognostic indicators and biomarkers that can evaluate H3K27M DMG’s response to imipridones. The H3K27M variant allele fraction (VAF) was measured in cell-free tumor DNA samples in DMG patients treated with ONC201. Decreased VAF levels correlated with increased progression-free survival, nearly doubling time to progression [133]. Interestingly, H3K27M VAF spikes preceded progression in a majority of cases [133]. As such, H3K27M VAF may serve as a potential prognostic indicator for imipridone therapy in DMG to predict progression.

Moreover, demonstrating the biochemical effects of imipridone therapy, it was reported that mice bearing orthotopic DMG xenografts treated with ONC206 accumulate gamma-aminobutyric acid (GABA) levels within a week of treatment [134]. Acting in an autocrine manner, it was further shown that GABA can mitigate imipridone-induced oxidative stress to avoid apoptosis. GABA seemed to be a unique compensatory adaptation to imipridones. The emergence of this GABA-mediated cytoprotection suggests that glioma cells may adapt to mitochondrial stress, underscoring the potential need for combination strategies [134].

A summary of clinical trials that employ imipridones in high-grade glioma is provided in Table 2.

## 7. Mitochondrial Targeting Can Alter the Epigenetic Landscape in Gliomas

**Figure 2:**
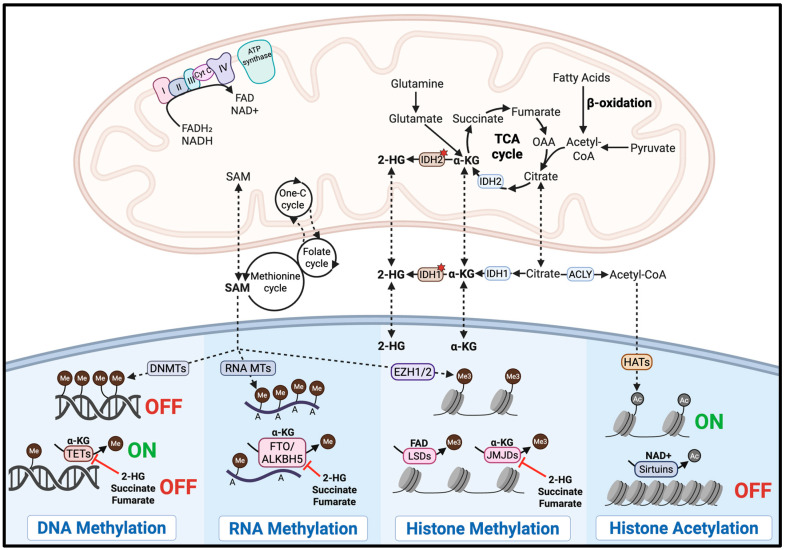
Mitochondrial-Epigenetic Crosstalk in Glioma. Mitochondrial metabolites are linked to the activity of epigenetic enzymes. The red stars indicate mutant IDH. The image was created with BioRender.com accessed on 2 September 2025.

Glioma exhibits strong pathogenic crosstalk between mitochondrial function and epigenetic regulation, which can be exploited through mitochondrial-targeting strategies. This interplay is mediated by key mitochondrial metabolites that serve as substrates or cofactors for epigenetic enzymes, as illustrated in Figure 2.

### 7.1. Mitochondrial-Epigenetic Crosstalk in Glioma

Mitochondria play a critical role in regulating epigenetic modifications by supplying key metabolites required for chromatin remodeling. For instance, histone acetylation through histone acetyltransferases (HATs) depends on acetyl-CoA, a metabolite primarily generated in the mitochondria, to promote gene activation. In glioblastoma, fluctuations in acetyl-CoA levels have been shown to regulate H3K27 acetylation at specific genomic loci, with ATP citrate lyase (ACLY)-dependent acetyl-CoA production driving oncogenic gene expression programs involved in cell adhesion and migration [136]. Conversely, histone deacetylation is mediated by histone deacetylases, including the NAD^+^-dependent sirtuin family, linking their activity directly to the mitochondrial metabolic state [137]. Therefore, targeting mitochondrial function can alter the availability of acetyl-CoA and NAD^+^, leading to epigenetic reprogramming that may suppress oncogenic pathways in glioma.

Furthermore, DNA, RNA, and histone methylation is regulated by DNA methyltransferases (DNMTs), RNA methyltransferases (RNA MTs), and histone methyltransferases, including enhancer of zeste homolog 1 and 2 (EZH1 and EZH2). All of these methyltransferases require S-adenosylmethionine (SAM) as the methyl group donor [138,139]. While SAM is primarily synthesized in the cytosol, its production depends on mitochondrial metabolism, which supplies one-carbon units via the folate and methionine cycles [137]. In glioma stem cells, methionine depletion and resulting reduction in SAM levels led to global DNA demethylation and altered histone methylation, ultimately decreasing the expression of stemness-related genes, impairing tumor growth and inducing cell death [140]. Thus, targeting mitochondrial function may reduce SAM availability, disrupt epigenetic regulation, and exert antitumor effects through epigenetic reprogramming.

DNA demethylation is mediated by ten-eleven translocation (TET) dioxygenases. RNA demethylation is performed by the fat mass and obesity-associated protein (FTO) and the AlkB homolog 5 protein (ALKBH5) [141,142]. Histone demethylation is carried out by JmjC-domain demethylases (JMJDs) and lysine demethylases (LSDs). TETs, FTO, ALKBH5, and JMJDs require α-ketoglutarate (α-KG) as a cofactor, a key intermediate of the TCA cycle in the mitochondria, whereas LSDs depend on FAD, another mitochondrial metabolite [137,138,142,143]. Therefore, targeting the mitochondria may reduce intracellular levels of α-KG or FAD, decreasing demethylase activity and thereby disrupting pathogenic gene expression programs in glioma.

Notably, oncometabolites such as 2-hydroxyglutarate (2-HG), succinate, and fumarate can competitively inhibit α-KG-dependent demethylases. In high-grade gliomas with the mitochondrial IDH2 mutation, mutant IDH2 aberrantly produce 2-HG, which inhibits TETs, FTO, ALKBH5, and JMJDs. This inhibition promotes widespread DNA hypermethylation and transcriptional silencing [144]. Targeting mitochondrial function may lower 2-HG levels and help reverse the epigenetic alterations driving oncogenesis in IDH-mutant gliomas.

Together, these metabolite–enzyme interactions underscore the critical role of mitochondrial metabolism in shaping the epigenetic landscape of glioma. By influencing the availability of key cofactors and substrates, mitochondria help regulate DNA, RNA, and histone modifications that control gene expression. These insights suggest that targeting mitochondrial function could potentially reverse pathogenic epigenetic alterations and suppress tumor growth. However, the epigenetic consequences of mitochondrial-targeted therapies in glioma remain largely unexplored, with limited direct evidence available to date.

### 7.2. Evidence for Epigenetic Modulation via Mitochondrial Targeting

The drug class of mitochondrial-targeting imipridones have demonstrated a remarkable ability to reverse the pathogenic phenotype of H3K27-altered DMG, which is characterized by global histone hypomethylation and a widespread reduction in H3K27me3, driving aggressive tumor growth. ONC201 can reverse the epigenetic state of DMG by increasing genomic H3K27me3 in H3K27M-DMG patient tumors. The early reversal of low H3K27me3 with ONC201 treatment was incredibly promising [135]. The investigators also observed a variety of favorable epigenetic alterations after treatment with ONC201, resulting from decreased chromatin accessibility at promoters and enhancers. By simultaneously suppressing key energy-producing metabolic pathways and reversing pathogenic reduction in H3K27me3, the treatment led to epigenetic downregulation of neuroglial differentiation and cell cycle genes in H3K27M-mutant DMG cells [135]. These epigenetic changes resulting from ClpP agonism suggest that targeting the mitochondria may also lead to epigenetic modulation, contributing to the therapeutic effect.

This connection has also demonstrated potential in other high-grade gliomas. A mitochondrial-targeting nanotherapy has been reported to exert potent antitumor effects in high-grade IDH2-mutated glioma by disrupting the mitochondrial–epigenetic axis. The hypericin-conjugated gold nanoparticles accumulate in glioma mitochondria and, upon red-light activation, induce oxidative stress and mitochondrial dysfunction, leading to a reduction in mutant IDH2 levels. In addition, degradation of the histone methyltransferases EZH1 and EZH2 led to decreased histone trimethylation, potentially reactivating tumor suppressor genes. Efficacy of this dual-targeting effect was demonstrated in xenograft mice models [145]. While epigenetic modulation via targeting the mitochondria in glioma remains highly unexplored, it holds potential in enhancing the therapeutic efficacy of mitochondrial targeting.

## 8. Conclusions

It is evident that the mitochondria are central orchestrators of glioma development, performing a wide array of dynamic functions that support rapid tumor growth. Through extensive metabolic reprogramming, glioma mitochondria supply the energy and biosynthetic intermediates required for glioma progression and resistance, establishing them as an attractive therapeutic target. Several preclinical and clinical efforts have aimed to exploit this vulnerability, though success has been mixed. A major clinical barrier has been toxicity, as demonstrated in the phase I trial of IACS-010759, which failed to reach the recommended phase II dosing due to neurotoxicity [78]. These challenges stemming from narrow therapeutic windows and systematic effects highlight the need for cautious advancement.

A precision medicine approach may represent the next logical step, leveraging mitochondrial-targeted therapies in gliomas that exhibit heightened mitochondrial dependence. Deeper understanding of glioma-specific mitochondrial biology will be essential to identify predictive biomarkers and design more selective targeting strategies [146]. Specific features such as altered mitochondrial transfer, mitochondrial dynamics, or dysregulated mitophagy, offer emerging avenues for intervention.

For example, since mitochondrial transfer has been implicated in glioma immune evasion, its inhibition may enhance responses to immunotherapy [45]. Similarly, as both mitochondrial transfer and BNIP3-mediated mitophagy are linked to chemoresistance, targeting these processes in combination with temozolomide may improve treatment efficacy [43,49]. More broadly, disrupting mitochondrial quality control could impair glioma cells’ ability to adapt metabolically and withstand radiation or chemotherapy. Given the strong association between mitochondrial dysfunction and therapy resistance, targeting mitochondrial metabolic pathways may also delay resistance and potentiate the standard-of-care, such as temozolomide and radiation [12].

Recently, the discovery of imipridones and induction of mitochondrial degeneration through ClpP agonism has sparked hope in the mitochondrial-targeting strategy, resulting in promising clinical trials for the treatment of DMG. The ability of imipridones to initiate mitochondrial collapse, trigger integrated stress response, and reverse epigenetic aberrations positions them as powerful, multifaceted agents, particularly in the context of DMG. Interestingly, combining ClpP agonists with epigenetic therapies, such as HDAC or EZH1/2 inhibitors, has also been shown to enhance antitumor efficacy in high-grade gliomas [147,148]. This new generation of imipridones hold promise in translating mitochondrial targeting into clinically meaningful outcomes.

Realizing the full therapeutic potential of mitochondrial targeting hinges on overcoming drug delivery challenges particularly across the blood–brain barrier (BBB) and into the mitochondrial compartment. Promising approaches include lipid-based nanocarriers, such as liposomes and solid lipid nanoparticles, engineered to cross the BBB and functionalized with mitochondrial-targeting moieties like triphenylphosphonium. Inorganic nanoparticles, including gold and mesoporous silica particles, can enhance intracellular delivery and be conjugated with mitochondrial-targeting ligands. Peptide-based systems, such as mitochondrial-penetrating peptides or mitochondrial targeting sequences, offer high specificity for mitochondrial membranes but often require integration with nanocarriers for effective blood–brain penetration. Collectively, advances in these platforms may help address the critical challenges of systemic toxicity while improving therapeutic specificity [149].

We acknowledge several limitations of this review. Firstly, our discussion of mitochondrial processes such as mitophagy and dynamics has been simplified to provide an overview of the current understanding. These processes are likely far more complex and context-dependent than represented here. Secondly, our discussion of drugs targeting mitochondrial respiratory complexes, including agents like metformin and arsenic trioxide, has been simplified. While these compounds exhibit mitochondrial activity, they often exert some antitumor effects through additional, non-mitochondrial mechanisms. Lastly, therapeutic strategies aimed at modulating epigenetics through mitochondrial targeting remain largely experimental. The current evidence base is limited, and further research is needed to validate the efficacy and mechanistic underpinnings of these approaches in glioma.

Looking ahead, the future of mitochondrial-targeted therapies in glioma will hinge on advancing our molecular understanding of glioma-specific mitochondrial vulnerabilities and on continued innovation in targeted drug delivery systems. As our insight into mitochondrial function in glioma deepens, so too does the potential to translate these insights into more precise, less toxic, and ultimately transformative therapies for patients with this devastating disease.

## Figures and Tables

**Table 1:** Clinical Trials employing OXPHOS inhibitors to treat High-Grade Glioma.

Mechanism of Action	Interventions	Clinical Trials (with NCT Identifier from ClinicalTrials.gov)	Phase	Status	Starting Year	Results
ETC Complex I inhibitor	Metformin	NCT05929495: Phase 2, Open-label, Single-arm Study on the Use of Metformin as Adjunctive Therapy in High-grade Glioma	II	Not yet recruiting	2023	No results posted.
		NCT05183204: Paxalisib with a High-Fat, Low-Carb Diet and Metformin for Glioblastoma	II	Recruiting	2022	No results posted.
		NCT04945148: Oxidative Phosphorylation Targeting In Malignant Glioma Using Metformin Plus Radiotherapy Temozolomide	II	Recruiting	2024	No results posted.
		NCT04691960: A Pilot Study of Ketogenic Diet and Metformin in Glioblastoma: Feasibility and Metabolic Imaging	II	Recruiting	2016	No results posted.
		NCT03243851: Study on Low Dose Temozolomide Plus Metformin or Placebo in Patient With Recurrent or Refractory Glioblastoma	II	Completed	2016	Although the metformin plus temozolomide regimen was well tolerated, it did not confer a clinical benefit in patients with recurrent or refractory GBM. The median OS was 17.22 months (95% CI 12.19–21.68 months) in the experimental group and 7.69 months (95% CI 5.16–22.67 months) in the control group, showing no significant difference by the log-rank test (HR: 0.78; 95% CI 0.39–1.58; *p* = 0.473) [74].
		NCT02780024: Metformin, Neo-adjuvant Temozolomide and Hypo-Accelerated Radiotherapy Followed by Adjuvant TMZ in Patients With GBM	II	Active, not recruiting	2015	The addition of metformin to the Adjuvant Temozolomide and hypofractionated accelerated radiotherapy regimen resulted in an encouraging improvement of the median OS compared with adjuvant temozolomide and hypofractionated accelerated radiotherapy alone. The main impact was observed in patients with unmethylated MGMT tumors [75].
		NCT02496741: Metformin And Chloroquine in IDH1/2-mutated Solid Tumors	I/II	Completed	2015	The treatment of advanced IDH1-mutated solid tumors with metformin and chloroquine was well tolerated but did not induce a clinical response in this phase Ib clinical trial [76].
		NCT02149459: Treatment of Recurrent Brain Tumors: Metabolic Manipulation Combined With Radiotherapy	I	Completed	2014	The intervention was fairly well tolerated; however, only moderate ketones levels were obtained. Metformin use and dietary intake were associated with higher serum ketone levels. The recommended phase II dose is the 8 weeks of a low-carbohydrate diet combined with 850 mg metformin twice daily [77].
		NCT01430351: Temozolomide, Memantine Hydrochloride, Mefloquine, and Metformin Hydrochloride in Treating Patients With Glioblastoma Multiforme After Radiation Therapy	I	Active, not recruiting	2011	Memantine, mefloquine, and metformin can be combined safely with TMZ in patients with newly diagnosed glioblastoma [73].
	IACS-010759	NCT03291938: IACS-010759 in Advanced Cancers	I	Completed	2017	IACS-010759 had a narrow therapeutic index with emergent dose-limiting toxicities, including elevated blood lactate and neurotoxicity, which obstructed efforts to maintain target exposure. Consequently, no recommended Phase II dose was established, only modest target inhibition and limited antitumor activity were observed at tolerated doses, and both trials were discontinued [78].
	IM156	NCT03272256: Phase 1 Study of IM156 in Patients With Advanced Solid Tumor and Lymphoma	I	Completed	2017	800 mg daily of oral IM156 was determined as the recommended Phase II dose. Observed adverse effects were manageable and stable disease was the best response [79].
ETC Complex IV inhibitor	Arsenic trioxide	NCT00720564: Radiation Therapy, Arsenic Trioxide, and Temozolomide in Treating Patients With Newly Diagnosed High-Grade Glioma	I	Completed	2008	Completed. No results posted.
		NCT00275067: Arsenic Trioxide, Temozolomide, and Radiation Therapy in Treating Patients With Malignant Glioma That Has Been Removed By Surgery	I/II	Completed	2005	Adding Arsenic Trioxide to radiotherapy and TMZ is feasible with no increased side effects. The addition of arsenic did not improve overall survival in glioblastoma patients as compared to historic data [89].
		NCT00185861: Phase I Trial of Arsenic Trioxide and Stereotactic Radiotherapy for Recurrent Malignant Glioma	I	Completed	2003	Arsenic Trioxide up to 0.25 mg/kg dose combined with stereotactic radiation is very well-tolerated for recurrent malignant glioma. Given the positive initial response and tolerability, phase II trials in patients with Malignant glioma are encouraged [96].
		NCT00095771: Arsenic Trioxide and Radiation Therapy in Treating Young Patients With Newly Diagnosed Gliomas	I	Completed	2004	Arsenic Trioxide in addition to radiation therapy was given to children with newly diagnosed anaplastic astrocytoma, glioblastoma, or diffuse intrinsic pontine glioma. Arsenic Trioxide was well tolerated throughout the entire dose escalation, resulting in confirmation of safety when administered 5 days per week during irradiation. The recommended dose of Arsenic Trioxide during conventional irradiation is 0.15 mg/kg given on a daily basis with each fraction of radiation therapy administered [97].
		NCT00045565: Arsenic Trioxide Plus Radiation Therapy in Treating Patients With Newly Diagnosed Malignant Glioma	I	Completed	2002	Arsenic Trioxide with standard radiation is well tolerated in patients with newly diagnosed glioblastoma. Even without temozolomide or adjuvant therapy, the overall survival of all patients (17.7 months) and especially patients who received biweekly ATO (22.8 months) is surprising and accompanied by pharmacodynamic changes on MRI. Further studies of this regimen are warranted [90].
ATP synthase/Complex V inhibitor	Gboxin	NCT06806228: Phase I Pilot Study to Evaluate the Anti-glioblastoma Effect of S-Gboxin in Standard Treatment of Glioblastoma/Diffuse Midline Glioma and Response to Treatment (Regardless of H3K27M Mutation Status)	I	Enrolling by Invitation	2025	No results posted.

**Table 2:** Clinical Trials employing Imipridones to treat High-Grade Glioma.

Mechanism of Action	Interventions	Clinical Trials (with NCT Identifier from ClinicalTrials.gov)	Phase	Status	Year Started	Results
ClpP Agonist	ONC201	NCT05580562: ONC201 in H3 K27M-mutant Diffuse Glioma Following Radiotherapy (the ACTION Study)	III	Recruiting	2023	No results posted.
		NCT05476939: Biological Medicine for Diffuse Intrinsic Pontine Glioma (DIPG) Eradication 2.0	III	Recruiting	2022	No results posted.
		NCT05009992: Combination Therapy for the Treatment of Diffuse Midline Gliomas	II	Recruiting	2021	No results posted.
		NCT04854044: ONC201 and Radiation Therapy Before Surgery for the Treatment of Recurrent Glioblastoma	I	Withdrawn (The P.I. is not prepared to move forward at this time)	2021	No results posted.
		NCT04629209: A Phase II, Open Label Study of ONC201 in Adults With EGFR-low Glioblastoma	II	Withdrawn (change in study approach)	2024	No results posted.
		NCT04617002: Intermediate-size Expanded Access to ONC201 for Patients With H3 K27M-mutant and/or Midline Gliomas	Expanded Access	Available	2020	No results posted.
		NCT03416530: ONC201 in Pediatric H3 K27M Gliomas	I	Terminated *	2018	Twenty-four of thirty patients were enrolled following radiation but prior to recurrence; six of thirty patients were enrolled with recurrent disease. Patient survival data was combined with data from NCT03134131, reporting a median OS of patients with non-recurrent H3K27M-DMG treated with ONC201 (*n* = 35) of 21.7 months from diagnosis and 9.3 months at recurrence [135].
		NCT03295396: ONC201 in Adults With Recurrent H3 K27M-mutant Glioma	II	Active, not recruiting	2017	No results posted.
		NCT03134131: Expanded Access to ONC201 for Patients With H3 K27M-mutant and/or Midline High Grade Gliomas	Expanded Access	No longer available	2017	Twenty-four of thirty patients were enrolled following radiation but prior to recurrence; six of thirty patients were enrolled with recurrent disease. Patient survival data was combined with data from NCT03416530, reporting a median OS of non-recurrent H3K27M-DMG patients treated with ONC201 (*n* = 35) of 21.7 months from diagnosis and 9.3 months at recurrence [135].
		NCT02525692: Oral ONC201 in Adult Recurrent Glioblastoma	II	Terminated *	2016	Among the 14 patients with recurrent disease prior to initiation of ONC201 treatment, median progression-free survival is 14 weeks, and the median OS is 17 weeks. Among the 4 pediatric patients enrolled, 2 DIPG patients remain progression-free for at least 53 and 81 weeks [116].
		NCT02038699: A First-in-man Phase I/II Study of Oral ONC201 in Patients With Advanced Cancer	I/II	Withdrawn **	2014	No results posted.
	ONC206	NCT04732065: ONC206 for Treatment of Newly Diagnosed, Recurrent Diffuse Midline Gliomas, and Other Recurrent Malignant CNS Tumors	I	Recruiting	2021	No results posted.
		NCT04541082: Phase I Study of Oral ONC206 in Recurrent and Rare Primary Central Nervous System Neoplasms	I	Recruiting	2020	No results posted.

* Terminated by an administrative protocol amendment. Decision to terminate the study was not related to any safety concerns of ONC201. ** Withdrawn prior to enrollment for unspecified reasons.
